# The Spi1/PU.1 transcription factor accelerates replication fork progression by increasing PP1 phosphatase in leukemia

**DOI:** 10.18632/oncotarget.16183

**Published:** 2017-03-14

**Authors:** Pauline Rimmelé, Michela Esposito, Laure Delestré, Jean-Hugues Guervilly, Maya Ridinger-Saison, Emmanuelle Despras, Françoise Moreau-Gachelin, Filippo Rosselli, Christel Guillouf

**Affiliations:** ^1^ Institut Curie, Paris, France; ^2^ Inserm U830, Paris, France; ^3^ Gustave Roussy Cancer Campus, Université Paris-Saclay, Villejuif, France; ^4^ Inserm U1170, Villejuif, France; ^5^ CNRS UMR8200, Equipe Labellisée La Ligue Contre Le Cancer, Villejuif, France; ^6^ CNRS, Paris, France

**Keywords:** DNA replication, leukemia, Spi1/PU.1 oncogene, CHK1, PP1

## Abstract

Oncogenes trigger replicative stress that can lead to genetic instability, which participates in cancer progression. Thus, determining how cells cope with replicative stress can help our understanding of oncogenesis and lead to the identification of new antitumor treatment targets. We previously showed that constitutive overexpression of the oncogenic transcription factor Spi1/PU.1 leads to pre-leukemic cells that have a shortened S phase duration with an increased replication fork speed and increased mutability in the absence of DNA breaks. Here, we demonstrate that the S phase checkpoint protein CHK1 is maintained in a low phosphorylation state in Spi1/PU.1-overexpressing cells and provide evidence that this is not due to negative control of its primary kinase ATR. Notably, we found that the expression of the CHK1 phosphatase PP1α is increased in Spi1/PU.1-overexpressing cells. By exogenously modulating its activity, we demonstrate that PP1α is required to maintain CHK1 in a dephosphorylated state and, more importantly, that it is responsible for the accelerated replication fork progression in Spi1/PU.1-overexpressing cells. These results identify a novel pathway by which an oncogene influences replication in the absence of DNA damage.

## INTRODUCTION

Cancer is the result of a multi-step process driven by the progressive accumulation of genetic and epigenetic changes in several genes that alter the activity and/or expression of their products, partners and targets. Thus, the safeguarding of genetic stability is a major defense against tumor initiation and progression. DNA damage, from cellular metabolism or exogenous sources, and unscheduled oncogene expression both lead to replicative stress and represent major sources of genetic instability.

Acute myeloid leukemia (AML) is a malignant disease that affects the myeloid lineage and progresses from normal to pre-leukemic and leukemic stages due to the accumulation of mutations over time. Although genomic alterations have been extensively characterized in AML [1], little is known about the determining events that favor the accumulation of mutations and the progression from the pre-leukemic to leukemic stage.

The transcription factor (TF) Spi1/PU.1 (herein referred as Spi1) is a primary regulator of hematopoiesis, as it is involved in the hematopoietic stem cell and progenitor self-renewal, as well as in myeloid and B lymphoid lineage commitment and maturation [2–5]. Similar to other differentiation-associated TFs, inappropriate Spi1 expression is oncogenic. However, the molecular mechanisms that mediate Spi1 oncogenic functions are complex and not fully understood. We have previously described a mouse model of erythroleukemia (a form of AML) initiated by Spi1 overexpression, which develops in several steps [6]. During the pre-leukemic stage, oncogenic Spi1 activity blocks erythroid progenitor differentiation [7] and promotes their survival by blocking apoptosis [7] by modulating the epigenetic control of the expression of the pro-apoptotic factor *Bim* [8]. Moreover, Spi1 overexpression reduces S phase duration and increases genetic instability by accelerating the speed of replication forks [9]. The leukemic stage is characterized by the emergence of malignant cells that have acquired *Kit* mutations, which promote the constitutive activation of several signaling pathways [6, 10]. Thus, we proposed a model in which Spi1 overexpression promotes cell transformation and contributes to leukemic progression by accelerating DNA replication, thus increasing the mutation load in pre-leukemic cells. Several oncogenes and tumor suppressors alter the replication program by affecting replication origin firing and causing the formation and accumulation of DNA strand breaks [11, 12]. Conversely, we have previously shown that Spi1 accelerates DNA chain elongation without affecting replication origin firing or promoting DNA strand break formation [13]. Interestingly, compared to other oncogenes or tumor suppressors, Spi1 induces a unique replicative stress, whose underlying mechanism remains elusive.

Here, we further investigated the mechanism by which Spi1 regulates DNA replication fork speed. We demonstrate that Spi1 overexpression in pre-leukemic cells is associated with increased expression of the phosphatase PP1α, which, in turn, is involved in maintaining CHK1 in a dephosphorylated and inactive form. Moreover, we demonstrate that PP1α is responsible for Spi1 shortening of S phase duration by increasing replication fork speed. Altogether, we identified a novel pathway through which an oncogenic transcription factor regulates DNA replication.

## RESULTS

### Spi1 overexpression reduces CHK1 phosphorylation in pre-leukemic cells

The checkpoint kinase CHK1 plays a major role in controlling S phase progression [14]. In unperturbed conditions, pharmacological inhibition or siRNA-mediated depletion of CHK1 increases replication origin firing and shortens S phase, demonstrating that CHK1 is essential for optimal DNA replication and is required to overcome spontaneous DNA replication difficulties [15–17].

**Figure 1:**
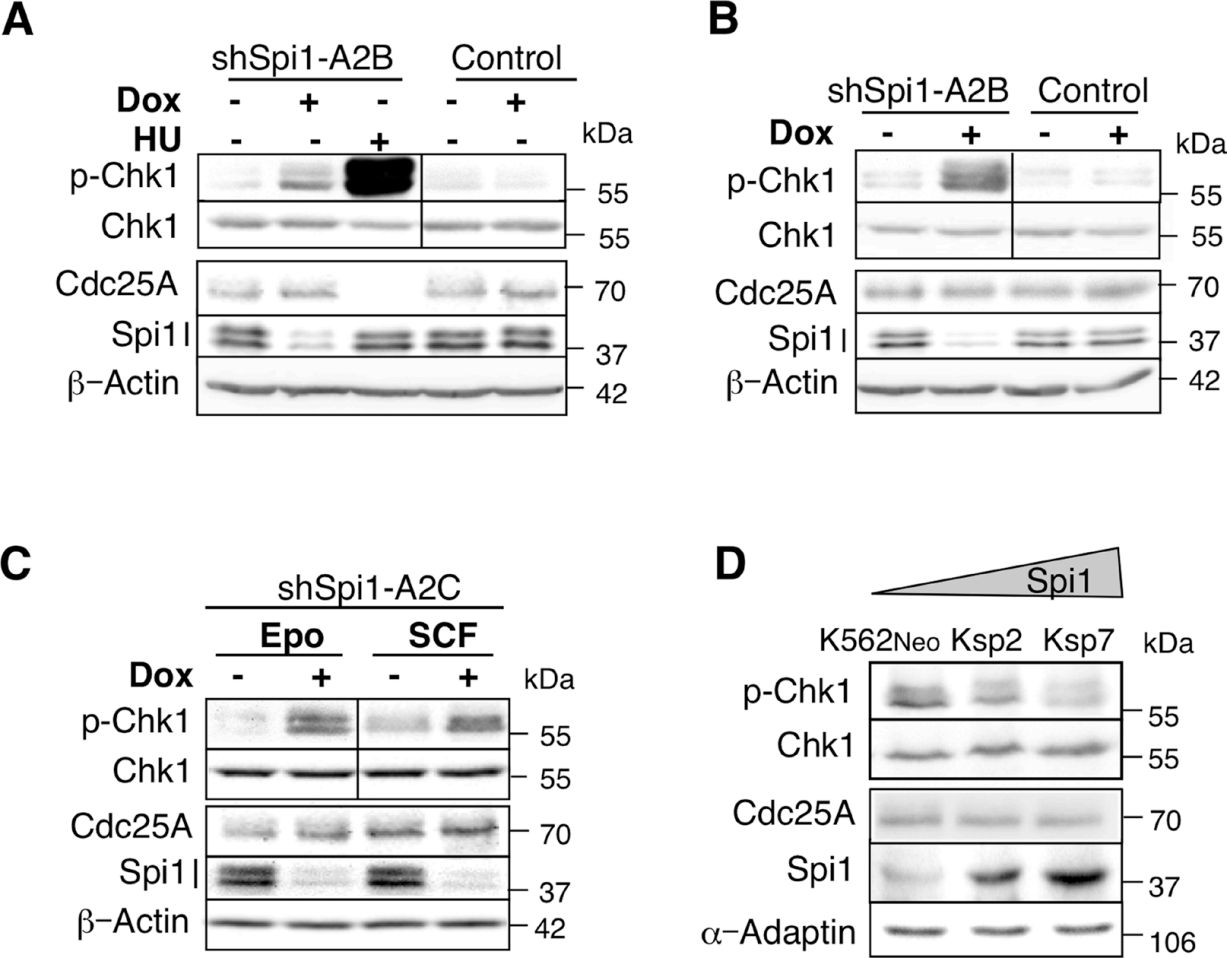
CHK1 phosphorylation in Spi1 pre-leukemic cells is increased following Spi1 down-regulation Whole-cell lysates from *Spi1* pre-leukemic cells (ShSpi1-A2B) or control cells cultured with Epo (**A**) or SCF (**B**) and treated with (+) or without dox (–) for 3 days to induce expression of anti-Spi1 siRNAs were analyzed by immunoblotting using anti-phosphoSer345 CHK1, anti-CHK1, anti-CDC25A, anti-Spi1 and anti-actin antibodies. The vertical bar on the p-CHK1 and CHK1 immunoblots separates cell samples that were analyzed on two separate membranes. HU: cells were incubated (+) or not (–) with 0.2 mM hydroxyurea for 2 h to induce ATR activity. (**C**) Whole-cell lysates from *Spi1* pre-leukemic cells (ShSpi1-A2C) cultured with Epo or SCF and incubated with dox (+) or not (–) for 3 days were analyzed by immunoblotting using anti-phosphoSer345 CHK1, anti-CHK1, anti-CDC25A, anti-Spi1 and anti-actin antibodies. (**D**) Protein extracts from K562 cells harvested after 24 h of culture were analysed by immunoblotting using anti-phosphoSer345 CHK1, anti-CHK1, anti-CDC25A, anti-Spi1 and anti-adaptin.

So, to determine whether CHK1 was involved in the acceleration of replication fork progression due to Spi1 previously described [13], its phosphorylation status was evaluated in cells expressing different levels of Spi1. First, we used pre-leukemic cells derived from the bone marrow of *Spi1* transgenic mice (called TgSpi1 cells herein) [7] in which Spi1 expression can be down-regulated by the expression of doxycycline (dox)-inducible shRNAs against *Spi1* (shSpi1-A2B and shSpi1-A2C cells) [9]. The presence of erythropoietin (Epo) or stem cell factor (SCF) is required for TgSpi1 cell proliferation. Dox-induced Spi1 down-regulation in both shSpi1-A2B and shSpi1-A2C cells was accompanied by a clear increase in CHK1 Ser345 phosphorylation (Figure 1). CHK1 phosphorylation was unchanged in control cells in which dox addition did not decrease Spi1 expression (Figure 1A and 1B). Importantly, we observed increased CHK1 phosphorylation in cells cultured in the presence of Epo, which allows erythroid differentiation (Figure 1A and 1C), or in the presence of SCF, which is not permissive for blast differentiation [13] (Figure 1B and 1C), indicating that CHK1 phosphorylation and erythroid differentiation are independent consequences of *Spi1* silencing. We have previously shown that Spi1 over-expression shortens S phase and increases replication fork speed in human K562 cells [13]. Likewise, in human K562 cells, increasing Spi1 expression resulted in decreased CHK1 phosphorylation (Figure 1D).

Altogether, these data show that Spi1 overexpression results in decreased CHK1 phosphorylation and increased replication elongation speed.

### PP1α phosphatase reduces CHK1 phosphorylation in Spi1-overexpressing cells

It has been reported that in human osteosarcoma and colon carcinoma, pharmacological or siRNA-mediated CHK1 inhibition leads to an increase in both the global replication rate and replication initiation and to a massive accumulation of DNA strand breaks [18]. We have observed that in both human and mouse hematopoietic cells, Spi1 overexpression is associated with down-regulation of CHK1 phosphorylation, an increase in the global replication rate and acceleration of the replication fork speed without modification of the replication initiation program. Importantly, the changes in replication by Spi1 do not induce DNA strand breaks [13].

**Figure 2:**
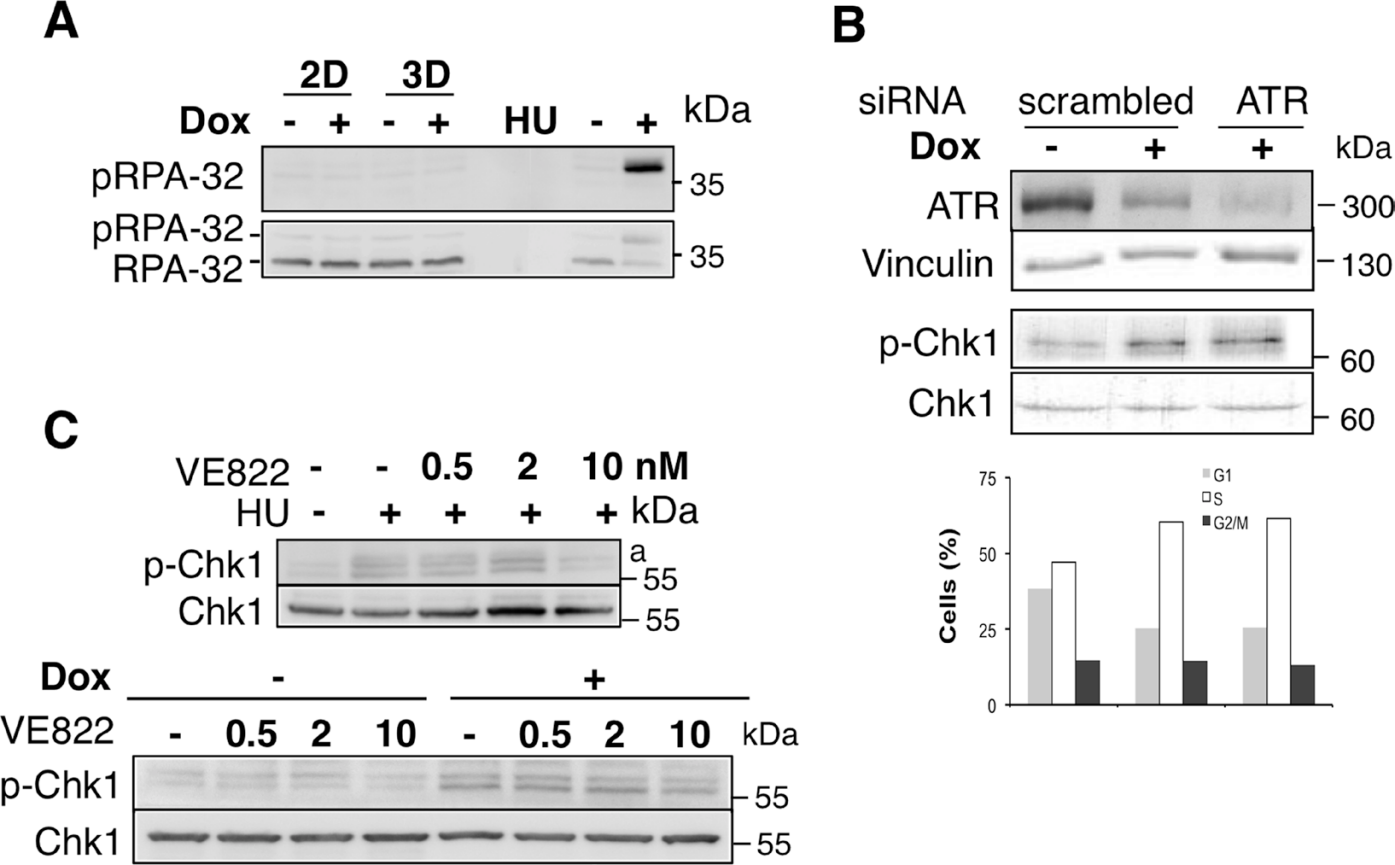
Reduced CHK1 phosphorylation in Spi1 pre-leukemic cells is not due to altered activity of DNA damage response kinases (**A**) Whole-cell lysates from *Spi1* pre-leukemic cells (ShSpi1-A2B) or control cells cultured with Epo and treated with (+) or without dox (–) for 2 (2D) or 3 days (3D) to induce expression of anti-Spi1 siRNAs were analyzed by immunoblotting using anti-phosphoRPA32 and anti-RPA32 antibodies. HU treatment (0.2 mM for 2 h) was used as positive control of RPA32 phosphorylation by ATR. (**B**) ShSpi1-A2B cells transfected with 100 nM of anti-ATR siRNA were cultured in the presence of dox and control cells (transfected with 100 nM of scrambled siRNA) were cultured with (+) or without dox (–) for 3 days. Medium was supplemented with Epo in both conditions. Whole-cell lysates were analyzed by immunoblotting using anti-ATR, anti-Vinculin, anti-phosphoSer345 CHK1 or anti-CHK1 antibodies. The histograms show the percentage of cells in G0/G1 (G1), S and G2/M (G2) phase from one representative experiment analyzed by flow cytometry. (**C**) ShSpi1-A2B cells were treated with HU (0.2 nM) and VE822 (2 and 10 nM for 2 h) or not (above pannel). ShSpi1-A2B cells were cultured in the presence (+) or not (–) of dox for 3 days and VE822 (2 and 10 nM for 2 h) were added or not to the cells, as indicated, 2 h before being harvested for analysis.

Consistent with the absence DNA breaks, RPA-32 protein, which is an activator and an early target of the primary CHK1 kinase ATR in the presence of single strand DNA regions [19, 20], was not phosphorylated in Spi1-overexpressing pre-leukemic cells, nor was it phosphorylated in cells with reduced Spi1 expression (treated with dox) (Figure 2A). In contrast, the replication inhibitor hydroxyurea (HU), which is typically used to induce ATR activity, triggers rapid RPA-32 phosphorylation, as expected [21]. These results indicate that, although ATR can be activated by inducers of replicative stress, basal ATR signaling was not modified by differential expression of Spi1. Moreover, siRNA knockdown of ATR did not reduce CHK1 phosphorylation or modify the cell cycle. Moreover even though the ATR inhibitor, VE-822 (Figure 2B), decreases slightly CHK1 phosphorylation, it did not abrogated the differences of CHK1 phosphorylation between Spi1-overexpressing and Spi1-knockdown cells, (Figure 2C). Notably, 10 nM of the ATR inhibitor was extremely efficient at inhibiting HU-induced CHK1 activation (Figure 2C). These data show that in a situation in which ATR is inactivated, Spi1-overexpressing cells still displayed a reduced CHK1 phosphorylation. Finally, we found that ATR expression increased in Spi1-overexpressing cells compared to dox-treated shSpi1-A2B cells (Figure 2B), revealing an inverse correlation between ATR expression and CHK1 phosphorylation. This correlation may be due to compensatory ATR expression in response to the maintenance of dephosphorylated CHK1 or to differences in the proportion of cell cycle phases and proliferation.

Altogether, these data are inconsistent with ATR being a major contributor to Spi1-mediated modulation of CHK1 phosphorylation.

**Figure 3:**
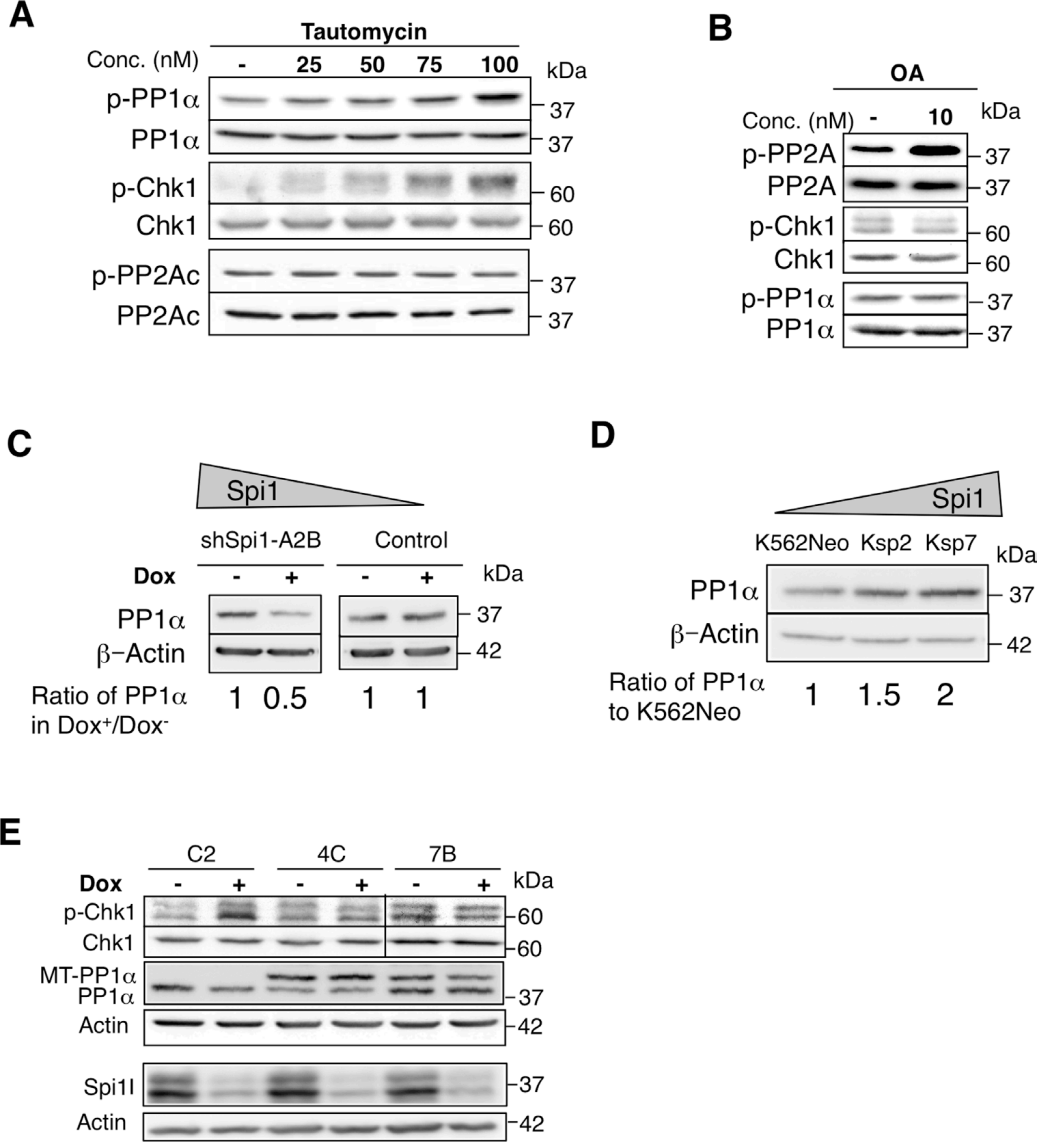
The PP1 phosphatase decreases CHK1 phosphorylation (**A** and **B**) ShSpi1-A2B cells were incubated with tautomycin (a PP1 inhibitor) (A) or okadaic acid (OA, a PP2A inhibitor) (B) at the indicated concentrations for 4 h. Whole-cell lysates were analyzed by immunoblotting using anti-phospho Thr320 PP1α, anti-PP1α, anti-phosphoSer345 CHK1, anti-phospho Tyr307 PP2Ac or anti-PP2Ac antibodies. (**C**) Whole-cell lysates from ShSpi1-A2B cells cultured with Epo and incubated (+) or not with dox (–) for 3 days and (**D**) K562 cells harvested after 24 h of culture were analyzed by immunoblotting using anti-PP1α and β-actin antibodies. Quantification relative to untreated pre-leukemic (dox-) or K562Neo control cells for equal protein levels based on the β-actin signal are indicated. (**E**) ShSpi1-A2B cells that stably express the Myc-tagged mouse PP1α catalytic subunit (MT- PP1α; clones 4C and 7B) and control cells (vector alone; C2 clone) were incubated (+) or not (–) with dox for 48 h. Cell lysates were immunoblotted with the indicated antibodies. Actin was used as a loading control.

Substrate phosphorylation depends on the concerted action of kinases and phosphatases. Having excluded a main role of ATR kinase in mediating Spi1-induced difference of phosphorylation of CHK1, we hypothesized that a phosphatase activity was responsible for the low CHK1 phosphorylation status in Spi1-overexpressing cells. It has been reported that PP1 and PP2A are two serine/threonine-protein phosphatases that act on CHK1 [22, 23]. To investigate their involvement in Spi1-dependent regulation of CHK1 phosphorylation, we incubated TgSpi1 cells with tautomycin (a PP1 inhibitor) or okadaic acid (OA, a PP2A inhibitor) (Figure 3A and 3B). We determined the efficiency and specificity of these inhibitors by monitoring the level of Thr320 phosphorylation on the PP1 catalytic subunit α (p-PP1α) [24] and of Tyr307 phosphorylation on the PP2A catalytic subunit (p-PP2A) by immunoblotting with appropriate antibodies [25]. Tautomycin induced a dose-dependent accumulation of inactive p-PP1α without any effect on p-PP2A (Figure 3A). Moreover, consistent with the idea that the status of CHK1 phosphorylation is co-regulated by kinase and phosphatase activities, tautomycin exposure also induced a dose-dependent accumulation of p-CHK1 in TgSpi1 cells. In contrast, efficient inhibition of PP2A with OA did not modify CHK1 phosphorylation (Figure 3B). These findings suggest that PP1 activity, but not PP2A activity, decreases CHK1 phosphorylation in pre-leukemic cells.

Since PP1 is able to control CHK1 phosphorylation in TgSpi1 pre-leukemic cells, we examined the level of expression of PP1 catalytic subunit α protein (PP1α) in Spi1-expressing cells. We found that the level of PP1α expression increased in TgSpi1 cells compared to cells with low Spi1 expression (two fold after 2 or 3 days of Dox treatment) (Figure 3C). Similarly, PP1α level was up-regulated by Spi1 proportionally to its expression in K562 cells (Figure 3D). Conversely, Spi1 down-regulation did not affect PP1β and γ, two other PP1 catalytic subunits (data not shown).

The finding that PP1α increased upon Spi1 overexpression and that PP1 inhibition restored CHK1 phosphorylation despite high Spi1 expression strongly supports the hypothesis that PP1 is a major determinant of the Spi1 effect on CHK1 phosphorylation. To further explore this idea, we examined whether PP1α overexpression affects the level of CHK1 phosphorylation in cells overexpressing Spi1. We overexpressed Myc-tagged PP1α (MT-PP1α) in TgSpi1 pre-leukemic shSpi1-A2B cells (Figure 3E). As expected, decreased Spi1 expression in TgSpi1 control cells expressing only the puromycin resistance gene (C2) reduced PP1α and increased phospho-CHK1 levels (Figure 3E). In contrast, decreased Spi1 after dox treatment in MT-PP1α-overexpressing cells (clones 4C and 7B) did not increase CHK1 phosphorylation, supporting the hypothesis that Spi1 modulates CHK1 phosphorylation by increasing PP1 activity.

To date, the mechanism by which Spi1 modulates PP1α is unknown. Indeed, it does not involve neither transcriptional regulation, nor proteosomal or lysosomal degradation in Spi1-overexpressing pre-leukemic cells (Supplementary Figure 1).

In conclusion, our data validate that PP1 is a major regulator of CHK1 phosphorylation and demonstrate that its expression is increased in Spi1- overexpressing cells.

### PP1 activity accelerates fork progression speed and shortens S phase duration in Spi1 pre-leukemic cells

**Figure 4:**
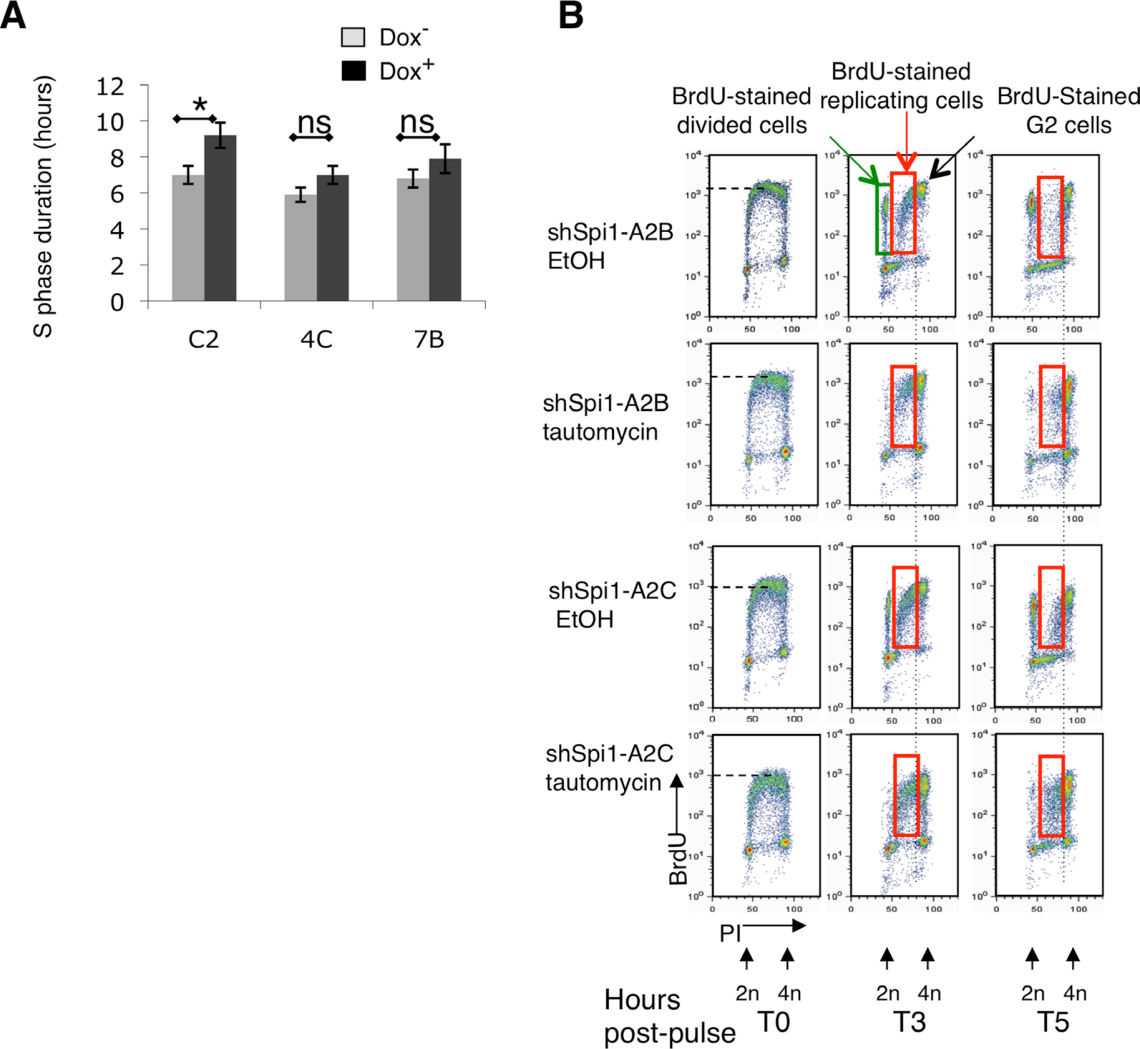
PP1α overexpression in Spi1 pre-leukemic cells inhibits CHK1 phosphorylation and the increase of S phase duration following Spi1 down-regulation (**A**) ShSpi1-A2B cells that stably express the Myc-tagged mouse PP1α catalytic subunit (MT- PP1α; clones 4C and 7B) and control cells (vector alone; C2 clone) were incubated (+) or not (–) with dox for 2 days. The histograms represent the mean ± SEM of three independent experiments of S phase duration measured using the relative movement method. Statistical differences were assessed with the Student's *t-test*: *P < 0.05. (**B**) S phase duration analysis by the relative movement method. ShSpi1-A2B and ShSpi1-A2C cells were incubated with 100 nM tautomycin (a PP1 inhibitor) or solvent alone (EtOH) for 4 h and pulse-labeled with BrdU for 15 min. Then, cells were fixed immediately (T0) or chased for 3 h (T3) or 5 h (T5) in the presence or not of tautomycin before analysis. The group of BrdU-positive dots between 2N and 4N corresponds to replicating cells in S phase (BrdU replicating). The group of dots at 2N represents the subsequent generation of cells in G1 (BrdU divided).

Since PP1 and is responsible for the low CHK1 phosphorylation status, we next examined whether PP1 affected S phase progression similarly to Spi1; i.e. by shortening S phase duration and accelerating the elongation speed. To answer this question, we first used the relative movement method that allows the evaluation of S phase duration by measuring the time that bromodeoxyuridine (BrdU)-labeled replicating cells in S phase take to enter the G2 phase [26]. As already published using this methodology [13], we found that Spi1 overexpression shortens S phase duration (Figure 4A, clone C2). Conversely, and consistent with our hypothesis, we found that the S phase duration was not significantly different between cells expressing or not Spi1 in MT-PP1α-overexpressing cells (Figure 4A, dox- versus dox+, clones 4C and 7B). Thus, PP1α seems to be a determinant of S phase duration.

We then performed the same experiment after tautomycin-mediated inhibition of PP1 activity. First, BrdU pulse-chase assays revealed that the overall level of nucleotide incorporation was lower in tautomycin-treated TgSpi1 cells than in cells treated with solvent alone (EtOH; control) (Figure 4B; BrdU fluorescence on Y axis), indicating a lower rate of nucleotide incorporation in cells in which PP1 activity was inhibited. Unfortunately, tautomycin treatment resulted in a strong G2 arrest, as deduced from the higher percentage of cells in G2 compared to solvent-treated cells at T0 (Figure 4B, 12% vs. 24% and 11% vs. 22% for shSpi1-A2B and shSpi1-A2C, respectively) and from the absence of BrdU-positive cells that had divided 3 h after the BrdU pulse (T3) (Figure 4B, green square, T3). Consequently, we were unable to calculate the precise length of S phase [26]. Nevertheless, tautomycin-treated samples clearly accumulated a higher number of BrdU-positive replicating cells at T3 and, more clearly at T5, compared to controls (see red squares in Figure 4B; 3 × 10^5^ total cells analyzed for each time point). This result indicates that the progression of cells into replication was delayed when PP1 activity was reduced by tautomycin.

In conclusion, PP1 inhibition slows down the progression of Spi1 pre-leukemic cells towards S phase, while PP1α overexpression has the opposite effect. These results show that PP1 is able to reduce CHK1 phosphorylation and accelerate S phase progression, even when Spi1 is weakly expressed, suggesting that it is the main regulator of S phase duration in pre-leukemic cells.

**Figure 5:**
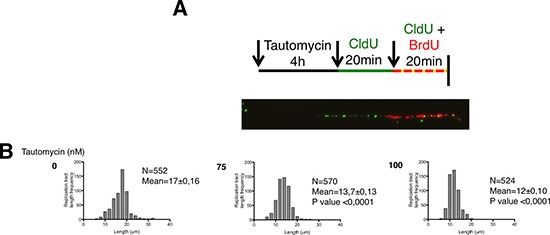
PP1 inhibition reduces the fork progression speed (**A**) Schematic representation of the experiments to measure replication fork speed. Types of DNA fibers scored in (**B**). Since CldU (green fluorescence) is not removed before the addition of BrdU (red fluorescence), DNA incorporating both analogous of nucleotides results in yellow/red fibers. (B) The distribution of DNA fiber lengths of CldU- and BrdU- labeled tracks in cells treated with and without tautomycin (0 nM, 75 nM, 100 nM) for 4 h. N, number of CldU and BrdU signal measurements; Mean fork speed ± SEM; *P value* by Mann-Whitney test relative to untreated (tautomycin 0 nM).

Thus, to definitively confirm the causal effect of PP1 in accelerating fork progression speed, we measured the elongation speed of replication by performing DNA fiber assays on tautomycin-treated TgSpi1 cells (Figure 5A and 5B). Asynchronous cells treated with 75 or 100 nM of tautomycin were sequentially pulse-labeled with CldU (green fluorescence) and BrdU (yellow fluorescence) for 20 minutes each, and cells were immediately harvested to measure fork kinetics. Representative images and the diagram of molecules taken into account are shown in Figure 5A. The means and distribution of the replication tract length were significantly (*P* < 0.0001) decreased in tautomycin-treated cells compared to untreated cells (Figure 5B), indicating a reduction in fork elongation speed when PP1 is inhibited and CHK1 is activated. Of note, both nucleotide analogues, CldU and BrdU, gave similar results (data not shown). Interestingly, the tautomycin-induced decrease in fork elongation was dose-dependent, as was CHK1 phosphorylation (Figure 3A).

In conclusion, our results demonstrate that in the presence of Spi1, PP1 maintains CHK1 in a low phosphorylation state and reduces S phase duration by accelerating fork progression.

## DISCUSSION

Oncogenes can be a source of genomic instability, and some of them act through replication stress [27, 28]. Having previously demonstrated that abnormal Spi1 expression triggers replicative stress [13], we aimed to identify its underlying mechanism(s) of action. Indeed, most oncogenes that have been studied in the literature cause replication stress by increasing origin firing and triggering DNA damage [12, 29], while Spi1 alters replication by increasing fork progression speed without altering origin firing. Furthermore, it does not generate DNA strand breaks but still increases the rate of gene mutations [13]. Thus, even though oncogene-induced replicative stress results in an increased proportion of cells in S phase [13, 30], the causes of this alteration might be different depending on the cellular context and oncogenes. Here, we provide evidence that the effect of Spi1 on replication is mediated by the PP1 phosphatase that also regulates CHK1 kinase phosphorylation, which is a central mediator of the S phase checkpoint.

Two functional assays using overexpression of the PP1α catalytic subunit or inhibition of its activity show that PP1 activity is sufficient and necessary to mediate Spi1 effect on acceleration of DNA replication and S phase duration. Importantly, we demonstrated that PP1α was differentially expressed according to Spi1 levels of expression, in TgSpi1 and K562 cells. Our results exclude transcriptional control of PP1α by Spi1 [31] or proteasome/lysosome-mediated PP1α degradation as mechanisms of PP1α modulation. Thus, how Spi1 controls PP1α steady state level remains an open question.

CHK1 has been shown to be deregulated as a consequence of ATR kinase inhibition by oncogenes involved in hematopoietic malignancies. For instance, BCL-6, an oncogene involved in B lymphoma, leads to reduced CHK1 phosphorylation through ATR inhibition, which subsequently facilitates DNA replication [32]. The oncogenic BCR-ABL fusion protein disrupts ATR-dependent intra-S phase checkpoint in chronic myeloid leukemia after etoposide treatment, by causing DNA strand break formation [33]. Several results argue against a defect in ATR kinase in Spi1-mediated decrease of CHK1 phosphorylation. Indeed, we found that decrease of ATR by siRNA in Spi1-silenced cells did not reduce CHK1 phosphorylation or the cell cycle. Moreover, pharmacological inhibition of ATR did not eliminate the Spi1-induced difference of CHK1 phosphorylation. Finally, the first target of ATR, namely RPA-32 was not activated in the pre-leukemic cells in both types of cells expressing or not Spi1. Interestingly, our findings show that Spi1-induced modulation of CHK1 phosphorylation status is due to PP1 phosphatase in murine and human cells. A role for PP1 in dephosphorylating CHK1 has been proposed for the recovery of DNA damage G2 checkpoint arrest in yeast [22]. Here, we demonstrate PP1 dephosphorylation activity on CHK1 Ser345 during an unperturbed cell cycle in pre-leukemic hyperproliferating cells.

CHK1 plays an essential role in the regulation of DNA replication. CHK1 prevents new origin firing by inhibiting CDK2 through CDC25A degradation, thus hindering the subsequent loading of CDC45 on chromatin to form the pre-initiation complex [34]. It has been reported that CHK1 inhibition slows replication fork progression associated with an increased origin firing in mammals [16, 17]. However, in response to camptothecin, inhibition of CHK1 accelerates both the speed of replication fork elongation and origin firing [35, 36]. Consequently, our results showing that PP1α overexpression causes the acceleration of replication fork speed and reduces CHK1 phosphorylation, led us to examine the role of CHK1 in the replication phenotype observed in Spi1-overexpressing cells. Unfortunately, our assay to define whether the lowest activity of CHK1 participates in accelerating fork elongation speed was unfruitful as CHK1 inhibition triggered a fast G1 phase arrest and cell death, which impeded successive analysis (data not shown). We can envision two possibilities: the reduction of CHK1 phosphorylation results in a global replication rate by favoring the speed of replication fork elongation or it does not play a role in the global acceleration of replication described in the Spi1-overexpressing cells. In that later case, other PP1α targets will be involved in fork progression acceleration by directly regulating cell cycle checkpoint [37], replication machinery [38, 39], chromatin structure [40] or the pool of nucleotides [41].

Recently, David and collaborators demonstrated that high CHK1 expression in AML is a marker of poor prognosis and resistance to cytarabine treatment. In that case, CHK1 inhibitors abolish cytarabine resistance and reduce the speed of fork progression in response to cytarabine [42]. Knowing the antagonistic effects of CHK1 in tumorigenesis [43], it is clear that more studies are required to understand the role of CHK1 and the effect of oncogenes at replication forks, that probably depend on the exogenous DNA damage inducers and on the nature of the cells.

Spi1-mediated acceleration of DNA replication in pre-leukemic cells may promote leukemic progression. We can envision at least two consequences, not mutually exclusive, of accelerated DNA replication in Spi1 pre-leukemic cells. Acceleration of replication may push cells through the cell cycle and sustain proliferation. In other words, it might provide cancer cells with a long-term proliferative advantage. Moreover, as we previously showed that Spi1 overexpression increases the mutation frequency in pre-leukemic cells, the accelerated replication rate may also promote leukemic progression by favoring the incidence of genomic instability [13]. This is particularly relevant in this leukemic model, as we have previously demonstrated the involvement of several oncogenic steps, which were associated with the acquisition of *de novo* genetic alterations [6].

## MATERIALS AND METHODS

### Mice and cell culture

TgSpi1 mice have been described previously [44]. Wild-type (WT) mice were obtained from crossing heterozygous TgSpi1 mice. Experiments were conducted with the ethical approval of Institut Gustave Roussy Area Standing Commitee on Animals. TgSpi1 cells and TgSpi1cells producing anti-*Spi1* shRNAs have been previously described [9]. Cells were grown in α-MEM supplemented with 5% fetal bovine serum (FBS) and 1 unit/mL erythropoietin (EPO) or 100 ng/mL stem cell factor (SCF). ShSpi1*-*A2B and ShSpi1*-*A2C cells produced two anti-Spi1 shRNAs in the presence of 100 ng/mL doxycycline. A total of 10^4^ cells/mL were used and parameters analyzed at the indicated times. Control cells expressed only TetR [9]. ShSpi1*-*A2B cells expressing the Myc-tagged mouse PP1α catalytic subunit were derived by cotransfection of the pEFBos-myc-PP1α vector (generous gift from Dorothée Buet) with the MSCV-puro vector. 4C and 7B are two puromycin-resistant clones (0.5 μg/mL) that overexpress PP1α. The C2 clone, which only expresses the puromycin resistance gene, was used as a control. Ksp2 and Ksp7 are K562 human leukemic cells in which Spi1 has been overexpressed, and were kindly provided by Dr. Delgado [45]. K562neo cells did not express exogenous Spi1. For all experiments, cells were diluted at 2 × 10^5^ cells/mL.

### Cell cycle and S phase analysis

For cell cycle analysis, cells were fixed in 70% cold ethanol and stained with propidium iodide (PI). S phase duration was calculated using the relative movement technique as described [26]. This technique estimates the S phase duration based on the amount of time that replicating cells in S phase spend before entering the G2-phase (4N). Briefly, cells were labeled with 30 μM BrdU (Sigma-Aldrich) for 15 min and fixed immediately. Alternatively, BrdU was washed out and cells were chased at 37°C for an additional 3 h or 5 h. Pelleted cells were resuspended in 30 mM HCl/0.5mg/mL pepsin. BrdU was immunodetected with a rat anti-BrdU antibody (Abcys) and a fluorescein-conjugated goat anti-rat antibody (Southern Biotechnology), and cells were stained with PI. Flow cytometry analyses were performed using FACSCalibur (Becton Dickinson, Meylan, France). Data were analyzed with the CellQuest Pro (Becton Dickinson, Meylan, France) and ModfitLT (Verity, Topsham, ME) software.

### Chemicals and siRNAs

Hydroxyurea (Sigma-Aldrich) was dissolved in water. MG132 (Sigma-Aldrich) was dissolved in DMSO. Bafilomycin (Sigma-Aldrich), tautomycin and okadaic acid (Calbiochem) were dissolved in EtOH. TARGETplus Smartpool siRNAs against ATR or control (non-targeting control pool-D-001810-10) were purchased from Dharmacon. Nucleofection with siRNAs was performed using the Amaxa nucleofector and the G16 program.

### DNA Fiber assay

Cells were successively labeled with 25 μM CldU and 75 μM BrdU for 20 min each. The DNA fiber spreads protocol was derived from Jackson and Pombo, 1998, with some modifications. Cells were harvested and resuspended in cold PBS1X. To prepare extended DNA fibers, 2 μL of cells (2 × 10^3^ cells) was spotted onto glass slides and lysed with 7 μl lysis buffer (0.5% SDS, 50 mM EDTA, 200 mM Tris-HCl). The microscope slide was carefully tilted to a 15° angle to allow spreading of the genomic DNA into single molecule DNA fibers by gravity. Fibers were then fixed in methanol and acetic acid (3:1) and subsequently acid treated with HCl (2.5 N) to denature the DNA fibers. Slides were neutralized and washed with PBS1X before blocking with 1% BSA and 0.1% Tween-20 in PBS1X for at least 1 h.

Slides were incubated for 1 h in blocking buffer containing primary antibodies against BrdU (mouse anti-BrdU Becton Dickinson, 1/20) and CldU (rat anti-BrdU Abcyss, 1/100). For the secondary antibodies, Alexa Fluor-594 goat anti-mouse IgG for the anti-BrdU antibody (Invitrogen Molecular Probes; 1/100) and Alexa Fluor-488 goat anti-rat IgG (Invitrogen Molecular Probes; 1/100) for the anti-CldU antibody were incubated for 30 min at 37°C. Then, DNA fibers were labeled with anti-DNA antibody, single stranded (Millipore, 1/100) for 45 min and the secondary and tertiary antibodies Alexa Fluor-350 rabbit anti-mouse and goat anti-rabbit IgG (Invitrogen, 1/100) for 20 min each. Slides were mounted in Mounting medium Dako (Invitrogen) prior to be analyzed using an Axio Imager Z1 microscope (Zeiss) and a 60× objective lens. Fluorescent fibers were captured, and the length of fluorescent signal was measured using Axiovision software (Zeiss).

### RNA extraction and quantification by real-time quantitative PCR

Total RNA was extracted using an RNeasy Mini kit (Qiagen), and PP1α expression was measured using a TaqMan expression assay as described previously [8].

### Immunoblotting and antibodies

The analysis of cell extracts by western blotting was performed as described previously [9]. For ATR analysis, 6% SDS-PAGE gels were used. Antibodies against phospho-CHK1 Ser345 and phospho-PP1α Thr320 were purchased from Cell Signaling; anti-ATR, anti- CHK1, anti-CdC25A, anti-PP1α and anti-PP2A antibodies were from Santa-Cruz Biotechnology; anti-RPA32 was from Genetex and phospho-RPA32 Ser33 was from Bethyl Laboratories. The anti-Vinculin and phospho-PP2A Tyr307 antibodies were purchased from Abcam. The β-actin antibody was purchased from Sigma-Aldrich and the anti-adaptin antibody from BD Biosciences. The anti-Spi1 antibody was described previously [9]. Specific peroxidase-conjugated secondary antibodies were used to detect protein expression using the LAS-3000 imager (Fujifilm). Images were cropped using Photoshop software (Adobe Systems France, Paris, France).

## SUPPLEMENTARY MATERIALS FIGURES


