# Supplementary material for: The Spi1/PU.1 transcription factor accelerates replication fork progression by increasing PP1 phosphatase in leukemia
Source: Oncotarget. 2017 Mar 14;8(23):37104–14. doi: 10.18632/oncotarget.16183 (PMC5514894; doi:10.18632/oncotarget.16183)
Supplement: Supplementary file 1 [file oncotarget-08-37104-s001.pdf]

The Spi1/PU.1 transcription factor accelerates replication fork progression by increasing PP1 phosphatase in leukemia

Supplementary Materials

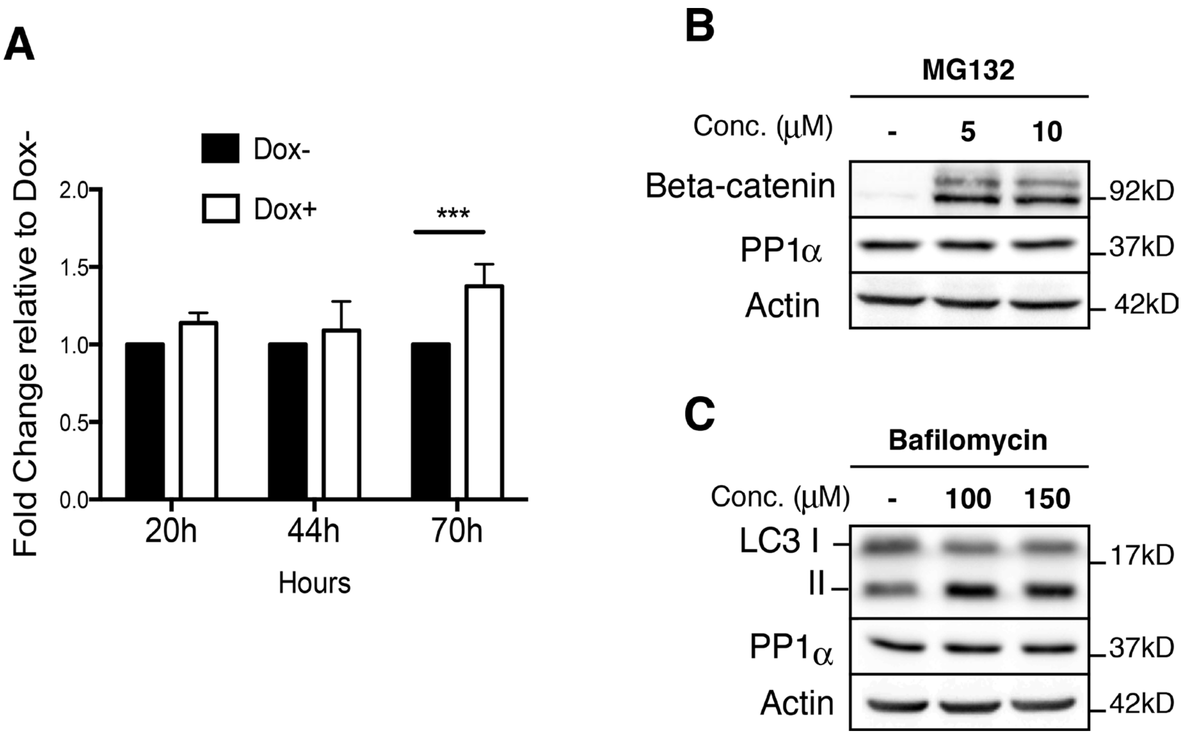

Supplementary Figure 1: Increase of PP1α associated with Spi-1 overexpression is not due to transcriptional modulation or protein stabilisation by Spi-1. (**A**) RNA was extracted from TgSpi1 pre-leukemic cells (ShSpi-1-A2B and ShSpi-1-A2C) cells cultured with Epo and treated with (+) or without (–) dox for 3 days to induce expression of anti-*Spi1* shRNAs. The expression of PP1α mRNA by real-time qPCR was normalized to Polr2α and to the level of untreated cells. The bars represent the mean ± SEM of three independent experiments. Two-tailed Student's t tests were used for statistical analysis. (**B** and **C**) Protein lysates from TgSpi1 pre-leukemic cells treated or not with MG132 (B) or with bafilomycin for 4h with the indicated concentrations were analyzed for the expression of PP1α and β catenin or LC3 as control of drug efficiency. β-actin served as the loading control.
